# Supplementary material for: Comparative genomics provides new insights into the diversity, physiology, and sexuality of the only industrially exploited tremellomycete: Phaffia rhodozyma
Source: BMC Genomics. 2016 Nov 9;17:901. doi: 10.1186/s12864-016-3244-7 (PMC5103461; doi:10.1186/s12864-016-3244-7)
Supplement: Additional file 6: — List of orphan genes with links to PFAM (related to Additional file 1: Table S1). (ZIP 1428 kb) [file 12864_2016_3244_MOESM6_ESM.zip › BLAST_HTML_FTR/G04057_P.html]

BLAST Search Results


```
BLASTP 2.2.27+


Reference:
Stephen F. Altschul, Thomas L. Madden, Alejandro A. Schäffer,
Jinghui Zhang, Zheng Zhang, Webb Miller, and David J. Lipman (1997),
"Gapped BLAST and PSI-BLAST: a new generation of protein database
search programs", Nucleic Acids Res. 25:3389-3402.


Reference for
composition-based statistics:
Alejandro A. Schäffer, L. Aravind, Thomas L. Madden, Sergei
Shavirin, John L. Spouge, Yuri I. Wolf, Eugene V. Koonin, and
Stephen F. Altschul (2001), "Improving the accuracy of PSI-BLAST
protein database searches with composition-based statistics and
other refinements", Nucleic Acids Res. 29:2994-3005.


Database: nr
           71,551,133 sequences; 26,053,659,533 total letters


Query= G04057_P

Length=316
                                                                      Score     E
Sequences producing significant alignments:                          (Bits)  Value

ref|XP_011455839.1|  PREDICTED: uncharacterized protein LOC105348...  42.0    0.98 
gb|EKC34950.1|  Myosin-10 [Crassostrea gigas]                         42.0    1.1  
ref|XP_012058189.1|  PREDICTED: solute carrier family 12 member 6...  40.4    2.4  
ref|XP_009221086.1|  hypothetical protein GGTG_05023 [Gaeumannomy...  39.3    5.7  
ref|XP_003136006.1|  hypothetical protein LOAG_00418 [Loa loa] >g...  38.9    7.7  


 >ref|XP_011455839.1| PREDICTED: uncharacterized protein LOC105348209 [Crassostrea 
gigas]
Length=6309

 Score = 42.0 bits (97),  Expect = 0.98, Method: Composition-based stats.
 Identities = 39/168 (23%), Positives = 83/168 (49%), Gaps = 17/168 (10%)

Query  144   KDREPDLSDEEKVMHE--GKRSDDVDENEDGEKVGEKSQAKN-----KLYERKLTESVLK  196
             +D +  + + EK+ HE   KR   ++E +  ++  EK++ K      + Y ++L +   +
Sbjct  5614  QDAKHSVEELEKLKHELEQKRKSQLEEAKKQQEEFEKNEKKRLEAEMEAYAKQLEKDTTR  5673

Query  197   EEE------DDLVEKRICSVEEKRVTLDEELERMIEFKVQTLLEEVMAGLRDELHERIVA  250
             E+E      + L +++   V+EK+  ++EELE++   + Q   E+    L ++ HER + 
Sbjct  5674  EKEQQDRKLEQLNKRKEDMVKEKKQKMNEELEKI---RQQGASEDEQKRLIEQ-HERDLQ  5729

Query  251   NLFVGMSVDTAMVHAFLAPQLKKLRSNIVHAVLMRSADGKEEKEREDE  298
             N+   M  D   + + L  +LKK +   +        +  +E+++E E
Sbjct  5730  NILNKMDADKMRMQSNLQERLKKKKDERLKNKQEELKENYKEQKKEME  5777


>gb|EKC34950.1| Myosin-10 [Crassostrea gigas]
Length=6274

 Score = 42.0 bits (97),  Expect = 1.1, Method: Composition-based stats.
 Identities = 39/168 (23%), Positives = 83/168 (49%), Gaps = 17/168 (10%)

Query  144   KDREPDLSDEEKVMHE--GKRSDDVDENEDGEKVGEKSQAKN-----KLYERKLTESVLK  196
             +D +  + + EK+ HE   KR   ++E +  ++  EK++ K      + Y ++L +   +
Sbjct  5579  QDAKHSVEELEKLKHELEQKRKSQLEEAKKQQEEFEKNEKKRLEAEMEAYAKQLEKDTTR  5638

Query  197   EEE------DDLVEKRICSVEEKRVTLDEELERMIEFKVQTLLEEVMAGLRDELHERIVA  250
             E+E      + L +++   V+EK+  ++EELE++   + Q   E+    L ++ HER + 
Sbjct  5639  EKEQQDRKLEQLNKRKEDMVKEKKQKMNEELEKI---RQQGASEDEQKRLIEQ-HERDLQ  5694

Query  251   NLFVGMSVDTAMVHAFLAPQLKKLRSNIVHAVLMRSADGKEEKEREDE  298
             N+   M  D   + + L  +LKK +   +        +  +E+++E E
Sbjct  5695  NILNKMDADKMRMQSNLQERLKKKKDERLKNKQEELKENYKEQKKEME  5742


>ref|XP_012058189.1| PREDICTED: solute carrier family 12 member 6 [Atta cephalotes]
Length=1122

 Score = 40.4 bits (93),  Expect = 2.4, Method: Composition-based stats.
 Identities = 38/148 (26%), Positives = 71/148 (48%), Gaps = 4/148 (3%)

Query  146   REPDLSDEEKVMHEGKRSDDVDEN-EDGEKVGEKSQAKNKL-YERKLTESVLKEEEDDLV  203
             +E +L+++E    E    D+  EN E+ + +G   +A+NK   E++  E+ +KE E+D+ 
Sbjct  971   QETELNNKEDSAEEANDKDEAKENNEETKLIGSSPKAENKENTEKEAKEAEVKETENDVQ  1030

Query  204   EKRICSVEEKRVTLDEELERMIEFKVQTLLEEVMAGLRDELHERIVANLFVGMSVDTAMV  263
             E +  S E K+ T+  +   +        L EV+  +  E  + ++ NL  G   DT M 
Sbjct  1031  ENKNQSPEIKKPTITPDEGDVRRMHTSLKLNEVIRKMSSEA-QLVILNL-PGPPRDTRME  1088

Query  264   HAFLAPQLKKLRSNIVHAVLMRSADGKE  291
                   +  ++ +  +  VLM    G+E
Sbjct  1089  RESNYMEFLEVLTEGLERVLMVRGSGRE  1116


>ref|XP_009221086.1| hypothetical protein GGTG_05023 [Gaeumannomyces graminis var. 
tritici R3-111a-1]
 gb|EJT79941.1| hypothetical protein GGTG_05023 [Gaeumannomyces graminis var. 
tritici R3-111a-1]
Length=1038

 Score = 39.3 bits (90),  Expect = 5.7, Method: Composition-based stats.
 Identities = 37/121 (31%), Positives = 47/121 (39%), Gaps = 11/121 (9%)

Query  12   LLLSDMPSSPGSRHSSVSVGRTGVRPKQHSTR--RATSIAVVPTVVPLLPAPKPRASADD  69
            L+L D+P  P   HS         +P+Q   R  R          +   P   PRAS D 
Sbjct  206  LILRDLPGDPAKEHSQEPASH---QPRQSEERQYRQEVQQPPQQPLQQTPMAMPRASNDG  262

Query  70   -VPTHSYSSPMKRPRVTASEESSKEKQSKGRRWTLSPMRRTISQTSFCGSDD----PLAD  124
             V +HS    M RPR  ASE         GR+ +    R      +F G DD    P+ D
Sbjct  263  YVGSHSVRPSMDRPR-NASETRRVSPTLVGRQSSSYHHRLDEKTQAFQGQDDMVEIPIVD  321

Query  125  D  125
            D
Sbjct  322  D  322


>ref|XP_003136006.1| hypothetical protein LOAG_00418 [Loa loa]
 gb|EFO28064.1| hypothetical protein LOAG_00418 [Loa loa]
Length=747

 Score = 38.9 bits (89),  Expect = 7.7, Method: Compositional matrix adjust.
 Identities = 33/110 (30%), Positives = 55/110 (50%), Gaps = 10/110 (9%)

Query  125  DAEDMLIIQCQDKVQEAYWKDREPDLSDE-EKVMHEGKRSDDVDENEDGEKVGEKSQAKN  183
            D +D+L     DK  +++ K  +  +  E +K++  GK  D + E E  +K G  S+ +N
Sbjct  168  DMQDLLKQNMSDKTLQSFRKKGKRKMQKEVQKIIRNGKIVDYLSEEEGSQKTGVDSKDEN  227

Query  184  KLYERKLTESVL--KEEEDDLVEKRICS----VEEKRVTLDEELERMIEF  227
                 +++E  +   EE+DD+   RICS     E+ R   D   ER I+F
Sbjct  228  FFETNEISEKNMGNGEEDDDV---RICSSFMDYEQGRAVKDTLCERFIKF  274


Lambda      K        H        a         alpha
   0.313    0.130    0.364    0.792     4.96 

Gapped
Lambda      K        H        a         alpha    sigma
   0.267   0.0410    0.140     1.90     42.6     43.6 

Effective search space used: 2543284270778


  Database: nr
    Posted date:  Sep 23, 2015 12:05 AM
  Number of letters in database: 26,053,659,533
  Number of sequences in database:  71,551,133


Matrix: BLOSUM62
Gap Penalties: Existence: 11, Extension: 1
Neighboring words threshold: 11
Window for multiple hits: 40
```
